# Doulas da morte: uma revisao de escopo*

**DOI:** 10.15649/cuidarte.2876

**Published:** 2023-12-25

**Authors:** Glenda Agra, Kádla Jocelli Gomes Rafael, Maria Heloyse de Lima Monteiro, Maria Aparecida Freire Avelar, Olavo Mauricio de Souza Neto, Tatiana Barbiere Santana

**Affiliations:** 1 Universidade Federal de Campina Grande - UFCG, campus Cuité -PB, Brasil. E-mail: g.agra@yahoo.com.br Universidade Federal de Campina Grande Universidade Federal de Campina Grande Cuité PB Brazil g.agra@yahoo.com.br; 2 Universidade Federal de Campina Grande UFCG, campus Cuité -PB, Brasil. E-mail. kadlajorceli@hotmail.com Universidade Federal de Campina Grande Universidade Federal de Campina Grande Cuité PB Brazil kadlajorceli@hotmail.com; 3 Universidade Federal de Campina Grande UFCG, campus Cuité-PB, Brasil. E-mail mariaheloysemonteiro@hotmail.com Universidade Federal de Campina Grande Universidade Federal de Campina Grande Cuité PB Brazil mariaheloysemonteiro@hotmail.com; 4 Universidade Federal de Campina Grande - UFCG, campus Cuité -PB, Brasil. E-mail: cida.avelar2014@gmail.com Universidade Federal de Campina Grande Universidade Federal de Campina Grande Cuité PB Brazil cida.avelar2014@gmail.com; 5 Universidade Federal de Campina Grande UFCG, campus Cuité-PB, Brasil E-mail: olavomauricio128@gmail.com Universidade Federal de Campina Grande Universidade Federal de Campina Grande Cuité PB Brazil olavomauricio128@gmail.com; 6 Fundado AmorTser. Rio Grande do Sul /RS. Brasil. E-mail: barbieretatiana@gmail.com Fundado AmorTser Rio Grande do Sul RS Brasil barbieretatiana@gmail.com

**Keywords:** Doulas Morte Assistencia Terminal, Doulas, Death, Terminal care, Doulas, Muerte, Cuidado Terminal

## Abstract

**Introdujo:**

O profissional doula da morte é considerado um colaborador solidário, cujo objetivo é melhorar a qualidade de morte no processo de terminalidade de pacientes, de forma a proporcionar uma ‘boa morte'.

**Objetivo:**

O objetivo do estudo é mapear as evidencias científicas sobre os papéis das doulas da morte na terminalidade da vida.

**Materiais e Métodos:**

Trata-se de uma revisao de escopo realizada nas bases de dados CINHAL, EMBASE, WOS, PUBMED e LILACS usando descritores de saúde conectados pelos operados booleanos AND e OR no espado temporal entre 2000 e 2021.

**Resultados:**

Dos 467 artigos encontrados, somente 11 fizeram parte da amostra final. Dentre a diversidade e flexibilidade de papéis, os profissionais doulas realizam tarefas, servidos e prestam cuidados práticos e nao clínicos durante todo o processo de morrer, morte, pós-morte e luto de pacientes e seus familiares, bem como promovem educado para a morte, levando em considerado as dimensóes biopsicossocial e espiritual do cuidado humano. Possíveis barreiras no movimento de doulas da morte incluem a inconsistencia nos programas de treinamento existentes e a ausencia de um órgao regulamentador para a supervisáo da prática e a padronizaáo de honorários.

**Discussao:**

O trabalho das doulas da morte concentra-se na presenta e atenáo plenas, escuta sensível, compassiva e centrada na pessoa em processo de terminalidade, respeitando os seus desejos e horando sua biografia. **Conclusao:** As doulas de morte podem aumentar os servidos de cuidados de fim de vida existentes, fornecendo servidos de cuidados holísticos e personalizados em todos os cenários da rede de atenáo a saúde, contudo, há necessidade de estudos mais rigorosos para explorar a percepáo dos profissionais de saúde sobre esse papel e investigar resultados clínicos entre pessoas que estáo morrendo e suas famílias.

## Abstract (en)

**Introduction:**

The professional death doula is considered a supportive collaborator, whose goal is to improve the quality of death in the process of terminality of patients, to provide a 'good death'.

**Objective:**

The objective of the study is to map the scientific evidence on the roles of death doulas in the terminality of life.

**Materials and Methods:**

This is a scoping review conducted in the databases CINHAL, EMBASE, WOS, PUBMED and LILACS using health descriptors connected by the Boolean operands AND and OR in the time frame between 2000 and 2021.

**Results:**

Of the 467 articles found, only 11 were part of the final sample. Among the diversity and flexibility of roles, professional doulas perform tasks, services and provide practical and non-clinical care throughout the dying, death, postmortem and bereavement process of patients and their families, as well as promote death education, considering the biopsychosocial and spiritual dimensions of human care. Possible barriers in the death doula movement include the inconsistency in existing training programs and the absence of a regulatory body for the supervision of practice and the standardization of fees.

**Discussion:**

The work of death doulas focuses on full presence and attention, sensitive, compassionate listening, and centering on the person in the process of terminality, respecting their wishes and honoring their biography.

**Conclusion:**

Death doulas can augment existing end-of-life care services by providing holistic and personalized care services across health care settings, however, there is a need for more rigorous studies to explore health professionals' perceptions of this role and investigate clinical outcomes among dying people and their families.

## Resumen

**Introducción:**

La doula de la muerte profesional se considera un colaborador de apoyo, cuyo objetivo es mejorar la calidad de la muerte en el proceso de terminalidad de los pacientes, con el fin de proporcionar una "buena muerte".

**Objetivo:**

El objetivo del estudio es mapear la evidencia científica sobre las funciones de las doulas de muerte en la terminalidad de la vida.

**Materiales y Métodos:**

Se trata de una revisión exploratoria realizada en las bases de datos CINHAL, EMBASE, WOS, PUBMED y LILACS utilizando descriptores de salud conectados por los operandos booleanos AND y OR en el marco temporal comprendido entre 2000 y 2021.

**Resultados:**

De los 467 artículos encontrados, sólo 11 formaron parte de la muestra final. Entre la diversidad y flexibilidad de roles, las doulas profesionales realizan tareas, servicios y proporcionan cuidados prácticos y no clínicos a lo largo del proceso de morir, muerte, postmortem y duelo de los pacientes y sus familias, así como promueven la educación sobre la muerte, teniendo en cuenta las dimensiones biopsicosociales y espirituales del cuidado humano. Entre los posibles obstáculos al movimiento de las doulas de la muerte se encuentran la incoherencia de los programas de formación existentes y la ausencia de un organismo regulador para la supervisión de la práctica y la normalización de los honorarios.

**Discusión:**

El trabajo de las doulas de la muerte se centra en la presencia y atención plena, la escucha sensible, compasiva y centrada en la persona en proceso de terminalidad, respetando sus deseos y honrando su biografía.

**Conclusión:**

Las doulas moribundas pueden aumentar los servicios existentes de atención al final de la vida proporcionando servicios de atención holísticos y personalizados en todos los entornos de atención sanitaria; sin embargo, se necesitan estudios más rigurosos para explorar las percepciones de los profesionales sanitarios sobre esta función e investigar los resultados clínicos entre los moribundos y sus familias.

## Introdujo

A palavra ‘doula' tem origem grega e significa ‘mulher que serve' e foi utilizada pela primeira vez na década de 1970 para designar mulheres que ofereciam apoio físico, emocional e suporte cognitivo a gestante, durante o parto e puerpério^1^^,^^4^. A partir do modelo das doulas de nascimento, pensou-se no desenvolvimento da formagáo de doulas de outras áreas especializadas^5^^-^^7^ dentre elas, as doulas da morte.

As doulas da morte surgiram pela primeira vez como cuidadoras e acompanhantes de pacientes em final de vida nos Estados Unidos, Reino Unido, Canadá e Austrália^8^^-^^12^. A doula da morte é alguém que acompanha, auxilia e apoia o paciente e familiares durante o processo de morte e morrer^2^^-^^4^^,^^8^.

Estudo mostrou que a discussáo da morte e a preparado dos cuidados voltados para o processo de morte de pacientes e seus familiares sáo insuficientes^13^. Além disso, esse mesmo estudo^13^ revelou que apesar de alguns pacientes poderem se beneficiar com os cuidados paliativos, eles apresentam cognigáo limitada no processo ativo de morte. Outro estudo 14 revelou que menos da metade dos pacientes em terminalidade relatou conhecer os cuidados em fim de vida. Isso mostra que as discussóes e as comunicares relacionadas a morte sáo frequentemente evitadas nas familias, uma vez que os familiares se sentem incomodados ou desconfortáveis a discutirem assuntos no entorno da morte.^15^^,^^16^

Nesta perspectiva, as doulas da morte podem preencher a lacuna nos cuidados de fim de vida, garantindo um continuum de cuidados as pessoas em terminalidade.^4^^,^^9^ Além disso, as doulas da morte estáo sendo apontadas como um facilitador que complementa a equipe de cuidados paliativos.^10^^,^^11^

Nesse cenário, o mapeamento científico sobre a definigáo, a importancia e as atividades das doulas da morte podem fornecer uma base abrangente de evidencias para colaborar na prática dos cuidados em fim de vida. Para tanto, utilizou-se o método de Revisáo de Escopo^17^, com o objetivo de mapear as evidencias científicas sobre os cuidados realizados pelas doulas da morte na terminalidade da vida.

## Materiais e Métodos

Foi utilizado o método Scoping Review, guiado por manual específico e sistematizado pela ferramenta PRISMA (PRISMA-ScR)^18^. A busca foi realizada por tres pessoas de forma independente, e posteriormente os resultados foram comparados. Os casos de dúvida foram resolvidos por consenso entre os pesquisadores.

Essa investigado baseia-se em cinco etapas: identificado da questáo de pesquisa; identificado dos estudos relevantes; selegáo dos estudos; análise dos dados; síntese e apresentado dos dados^19^. Enfatiza-se que todas as informales foram armazenadas no Mendeley^20^.

### Identificado da questáo da pesquisa

A questáo de pesquisa, o objetivo do estudo e os descritores foram elucidados pela combinado mnemónica PCC: P Population - doulas da morte; C Concept - cuidados em fim de vida; C Context - assistencia terminal. E apresentou a seguinte questáo norteadora: quais as evidencias científicas sobre os cuidados realizados pelas doulas da morte na terminalidade da vida?

### Estratégia de busca

Para identificado de estudos relevantes, foram selecionados artigos publicados em periódicos online no período de 1° de janeiro de 2001 a 31 de dezembro de 2021.

A estratégia de busca de artigos foi norteada pelos Descritores de Ciencias da Saúde (DeCs) e termos do Medical Subject Headings (MeSH) apresentados nos idiomas ingles, espanhol e portugués, com o auxílio do booleano AND e OR entre os seguintes termos: “doulas”; “morte”; “assistencia terminal”. Vale ressaltar, que a busca foi realizada de forma independente por tres pesquisadores. Salienta-se que nas fontes de busca nao foram obtidas publicares com os termos em portugués.

Foram utilizadas as seguintes bases de dados e bibliotecas eletronicas como fontes de informado: Web of Science (WOS), Cumulative Index to Nursing and Allied Health Literature (CINAHL), EMBASE, Literatura Latino-Americana e do Caribe em Ciencias da Saúde (LILACS), US National Library of Medicine National Institutes of Health (PUBMED).

De modo consequente, procedeu-se a comparado dos registros entre os tres avaliadores, com o intuito de dirimir dúvidas acerca da permanencia desses estudos.

### Seleáo dos Estudos

A selegáo dos estudos foi realizada a partir da leitura criteriosa dos resumos e títulos dos registros obtidos nas fontes de informado. Por conseguinte, após a análise dos textos completos, foram selecionadas as publicares a serem mapeadas em conformidade com os elementos PCC.

### Critérios de Inclusáo

Foram considerados estudos originais, revisóes, relatos de experiencia e de caso e editoriais. Foram excluídos, sites, blogs, notícias, informativos, artigos de revistas náo científicas e de jornais, resumos de congressos, notas prévias, dissertagóes, teses e artigos publicados em outros idiomas, indisponíveis na íntegra no momento da busca ou que náo apresentaram relagáo com o tema abordado.

### Extraáo dos Dados

Utilizou-se um roteiro elaborado pelos autores, onde os dados relevantes das publicagóes foram consolidados por tres revisores e extraídos de acordo com os objetivos desta revisáo. De modo sequencial, houve a associagáo das principais informagóes selecionadas a partir de uma reuniáo analítica e consensual com um quarto revisor.

## Resultados

**Figura 1:**
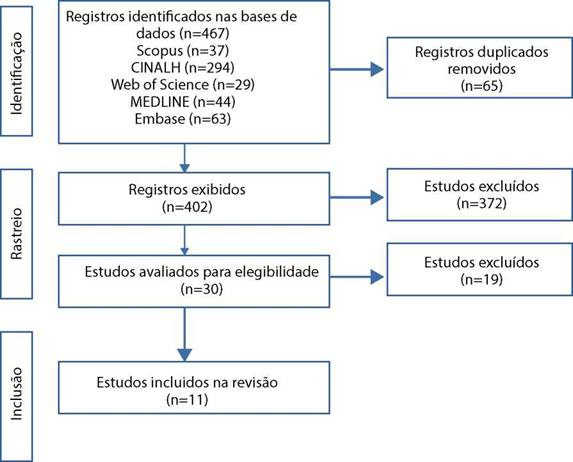
Diagrama PRISMA adaptado - ScR das publicares científicas selecionadas e incluidas na revisao de escopo

No total foram encontrados 467 artigos após a leitura dos títulos e dos resumos; destes, 30 foram excluídos por serem duplicados, totalizando 402 estudos. Após leitura flutuante, foram excluídos 372 artigos, uma vez que se tratava de estudos sobre as doulas do nascimento, restando 30 estudos. Após a leitura na íntegra, somente 11 artigos versavam sobre a temática e que sáo a amostra final por contemplar os critérios de inclusáo e exclusáo pré-estabelecidos. O processo detalhado da pesquisa e selegáo dos estudos desta revisáo está exposto no fluxograma (Figura 1) segundo indicagóes do JBI,concordante com o checklist adaptado do *Preferred Reporting Items or Systematic Reviews and Meta Analyses* (PRISMA).

Os estudos mencionados nesta revisao foram sintetizados no Quadro 1

**Quadro 1:** Síntese dos estudos conforme ano, autoria, periódico, título, país do autor principal, tipo de publicado, desenho do estudo, pessoas aptas e cenários laborativos internet, 2022 (n = 11)

N°	Ano	Autoria	Periódico	Título	País	Desenho do estudo	Pessoas	Cenários laborativos
1	2011	Corporon, K^21^	Baylor University Medical Center Proceedings	Comfort and caring at the end of life: Baylor's Doula Program	EUA	Editorial	Enfermeiros, membros da equipe de paliativos, capelao e terapeuta cuidados	Hospitais, domicílios, instituÍQóes de longa permanencia
2	2017	Fukuzawa, RJ; Kondo,KT^9^	Int J Palliat Nurs	A holistic view from birth to the end of life: end-of-life doulas and new developments in end-of-life care in the West	Japao	Revisao narrativa	Médicos, enfermeiros, cuidadores, voluntários	Hospitais, domicílios, casas de apoio, institutes de longa permanencia
3	2019a	Rawlings, D et al.^2^	Health Soc Care Community	Compassionate Communities. What role do Death Doulas play in end-of-life care? A systematic review	Australia	Revisao Sistemática	Enfermeiros, médicos e equipe de cuidados paliativos	Hospitais, domicílios, comunidades compassivas.
4	2019b	Rawlings, D et al ^3^	Health Soc Care Community	The voices of death doulas about their role in end-of-life care	Australia	Estudo quanti- qualitativo	Enfermeiros, médicos, parteiras, cuidadores e cuidadores de idosos.	Hospitais, domicílios, comunidades compassivas, agencias funerárias, death cafés
5	2020	Krawczyk, M; Rush, _M._ ^10^	Palliat Care Soc Pract	Describing the end- of-life doula role and practices of care: perspectives from four countries	Reino Unido	Estudo qualitativo	Enfermeiros, psicólogos, assistentes sociais, terapeuta holístico, agente funerário.	Hospices, hospitais, institutes de longa permanencia,domicílios, comunidades compassivas, death cafés
6	2021	Gaspard, G; Gadsby, C; Mallmes, J^22^	Int J Indig Heath	Indigenous End-of-Life Doula Course: Bringing the Culture Home	Canadá	Relato de experiencia	Qualquer pessoa que queira aprender como facilitar uma abordagem paliativa.	Comunidade indígena
7	2021	Maloon, A^23^	Palliat Care Soc Pract	Compassionate community structure and function: a standardized micro model for end-of-life doulas and community members supporting those who wish to die at home	Australia	Estudo qualitativo	Qualquer pessoa	Comunidades compassivas; hospitais particulares e públicos, domicílios, hospices, institutes de longa permanencia.
8	2021	Rawlings, D et al.^11^	Health Soc Care Community	End-of-life doulas: A qualitative analysis of interviews with Australian and International death doulas on their role	Australia	Estudo qualitativo	Enfermeiro, Assistente Social, Nutricionista, Psicoterapeuta, Cuidador, Cuidador de idosos, Capelao, Advogado, Administrador, Agente funerário	Domicílios, Hospitais e
9	2021	Rawlings, D; Davies, G; Tieman, J^4^	Public Heath	What does this mean for roles such as a death doula in end-of-life care?	Australia	Revisao narrativa	Médicos, enfermeiros, psicólogos	Comunidades Comunidades compassivas
10	2021	Dellinger, A; Husain, J. ^24^	Omega - J Death Dying	End-of-Life Doulas: Documentin Their Backgrounds and Services	EUA	Estudo quanti- qualitativo	Profissionais da área da saúde e pessoas treinadas	Hospices e/ou quaiquer outros tipos de servidos de saúde
11	2021	Francis, A.A. ^25^	J Contemp Ethnograf	Gender and legitimacy in personal service occupations: the case of End-of-Life Doulas and Death Midwives	EUA	Estudo qualitativo	Qualquer pessoa, assistentes sociais, enfermeiros, doulas do nascimento, psicoterapeuta, agente funerário, monge budista, cuidador da saúde, capelão.	Hospices, hospitais, domicíliose presídios.

A fim de facilitar o entendimento dos cuidados realizados pelas doulas da morte, foi elaborado o Quadro 2, que descreve as tarefas e os servidos fornecidos pelas doulas abrangendo as dimensóes do cuidado humano, numa perspectiva integral e holística do processo de morte e morrer.

**Quadro 2:** Tarefas e servidos das doulas da morte, conforme as dimensóes física, emocional/psíquica, social, espiritual e informativa/educacional, internet, 2022 (n = 11)

Dimensóes	Tarefas e Servidos
Física	Planejam antecipadamente todos os cuidados necessários para todo o processo de morrer, morte e de luto ^3^, ^10^, ^11^, ^24^, ^25^; Avaliam os sinais e sintomas do paciente ^3^, ^10^, ^24^, ^25^;
	Realizam medidas de conforto: ^3^, ^9^, ^11^, ^21^massagens ^2^, ^3^, ^9^, ^11^; termoterapia e/ou crioterapia^10^;
	Auxiliam nos cuidados físicos: na alimentado ^2^,^3^,^9^ na mudanza de decúbito ^3^,^9^,^23^, ^25^; nas higienes oral ^9^, corporal ^9^, íntima^9^ e pessoal^2^, ^3^,^23^, ^25^; na hidratado da pele ^2^,^3^,^23^; na deambulado ^2^,^3^,^21^, ^23^; nas necessidades excretórias ^9^,^22^; Administram medicamentos prescritos, desde que devidamente treinadas e autorizadas pela família^2^, ^3^, ^10^; Acompanham a pessoa durante a morte ^2^^-^^4^, ^9^^-^^11^, ^24^, ^25^;
	_Promovem vigília_^3^, ^4^, ^9^, ^10^, ^11^, ^23^, ^24^, ^25^_;_
	Realizam cuidados com o corpo pós-morte em domicílio:^2^, ^3^, ^10^, ^11^, ^24^, ^25^ higienizar^2^, tamponar ^10^, vestir^2^; preparar o corpo para o velório^2^.
Emocional/Psíquica	Promovem apoio emocional a pessoa que está morrendo e a sua familia ^2^,^3^,^10^, ^23^, ^24^;
	Realizam escuta ativa e empática ^21^,^23^^,^^25^; presenta compassiva ^3^^,^^21^^,^^23^^,^^23^ bem como usam estratégias de comunicado _empática_^3^, ^10^, ^21^, ^23^
	Apoiam a equipe multiprofissional e a familia ^3^^,^^10^^,^^11^^,^^23^^,^^25^ na comunicado do diag nóstico ao paciente^10^;
	Escutam queixas, preocupaqóes, angústias e temores do paciente e familiares ^2^,^3^,^10^, ^23^, ^25^;
	Acolhem emoqóes e sentimentos do paciente ^23^, ^5^ e seus familiares ^23^, ^25^, durante o diagnóstico^10^, o processo ativo de morte ^10^^); (^^24^, ^25^_, o pós-morte_^10^, ^24^_e o luto_^10^, ^24^_;_
	Estimulam a participado de familiares e de amigos durante todo o processo de morte ^10^, ^23^, ^24^, de forma a proporcionar dignidade nos últimos dias de vida do paciente, bem como período de descanso ao cuidador principal ^23^;
	Estimulam rodas de conversas com amigos a fim de contarem histórias e experiencias de vida ^2^,^3^,^10^, ^23^, ^25^que tiveram juntos;
	Oferecem um conjunto específico de habilidades, em particular, o tempo pessoal ^3^, ^10^, ^11^, ^23^, para fazer companhia ^3^, ^10^, ^11^, ^23^, ^25^; para ouvir^10^^,^^23^e apoiar na tomada de decisao ^2^^-^^4^, ^9^, ^10^, ^21^, ^23^, ^25^; respeitam os desejos ^3^, ^10^, ^23^, ^25^ e defendem os interesses do paciente ^2^, ^3^, ^10^, ^23^, ^24^ e da família durante todo o processo de morte e morrer ^3^, ^10^, ^11^, ^23^, ^25^;
	Realizam atividades em conjunto com o paciente: assistem a filmes e/ou a televisao^1^; leem livros ^10^, ^23^; cantam músicas^10^, ^11^; tocam algum instrumento ^10^, ^23^; fazem oraóes e/ou preces juntos ^4^
	Realizam práticas integrativas e complementares: musicoterapia^3^^,^^21^,; massoterapia^3^^,^^4^^,^^10^^,^^11^, reflexologia podal^10^, arteterapia^3^; aromaterapia^10^^,^^24^; meditado^4^; Reiki^4^,^11^^,^^24^; terapia assistida por animais^23^;
	Conduzem o paciente a relembrar momentos de vida, de forma a resgatar o sentido e o significado para o momento atual ^10^,^23^,^24^_;_
	Promovem apoio no luto aos familiares, aos amigos e a equipe de saúde do hospital após o óbito do paciente^2^^,^^3^^,^^10^^,^^11^^,^^24^
Social	Promovem apoio emocional a pessoa que está morrendo e a sua familia ^2^, ^3^, ^10^, ^23^, ^24^;
	Realizam escuta ativa e empática^3^^,^^21^^,^^23^^,^^25^; presenna compassiva^3^^,^^21^^,^^23^^,^^25^; bem como usam estratégias de comunicanao _empática_^3^^,^^10^^,^^21^^,^^23^_:_
	Apoiam a equipe multiprofissional e a familia^3^^,^^10^^,^^11^^,^^23^^,^^25^ na comunicado do diagnóstico ao paciente^10^;
	Escutam queixas, preocupares, angústias e temores do paciente e familiares^2^^,^^3^,^23^^,^^25^
	Acolhem emooes e sentimentos do paciente^23^^,^^25^ e seus familiares^23^^,^^25^, durante o diagnóstico^10^, o processo ativo de morte ^10^^,^^24^^,^^25^_, o pós-morte_^10^^,^^24^_e o luto_^10^^,^^24^_;_
	Estimulam a participado de familiares e de amigos durante todo o processo de morte10,23,24, de forma a proporcionar dignidade nos últimos dias de vida do paciente, bem como periodo de descanso ao cuidador principal ^23^;
	Estimulam rodas de conversas com amigos a fim de contarem histórias e experiencias de vida 2, 3, 10, 23, 25que tiveram juntos;
	Atuam como elo entre a pessoa que está morrendo e a familia^2^^,^^3^^,^^10^^,^^23^^-^^25^;
	Organizam e planejam a agenda semanal dos pacientes^23^^-^^25^;
	Acompanham pacientes que nao tem apoio familiar^1^, ou, que se sentem solitários ou abandonados^21^, ou, cujos cuidadores estao exaustos^21^^,^^23^^)^ e necessitam de descanso^3^^,^^10^^,^^21^^,^^23^;
	Acompanham o paciente em consultas médicas^2^^,^^3^^,^^10^^,^^11^^,^^23^^-^^25^; Transmitem a equipe médica queixas verbais e atitudes nao verbais adotadas pelo paciente^3^^,^^9^^,^^10^^,^^23^;
	Realizam ligares telefónicas para os profissionais de saúde da equipe médica, de forma a facilitar a comunicando entre a familia e o paciente^2^^,^^3^^,^^10^, ^23^^-^^25^;
	Mantém paciente e familiares informados sobre questoes médicas ^3^^,^^10^^,^^11^^,^^23^^-^^24^; progressao da doenna^3^^,^^23^; e processo ativo _de morte_^10^^,^^23^^-^^25^_;_
	Auxiliam o paciente nas atividades administrativas^2^^,^^3^^,^^11^^,^^23^^,^^24^: digitam e enviam e-mails^2^^,^^3^; recolhem correspondencia^23^; Acompanham^3^^,^^10^^,^^11^^,^^23^^-^^25^ e/ou proporcionam momentos de lazer ao paciente^9^^,^^23^;
	Facilitam a concretizanao dos desejos de fim de vida: algum momento festivo, como aniversário, casamento etc.^2^^,^^3^^,^^23^^,^^25^; Organizam documentos juridico-legais em conjunto com os profissionais envolvidos (por ex: certidao de óbito)^10^^,^^24^; Auxiliam a familia no planejamento do funeral (por ex: cerimónia do memorial, velório e sepultamento ou cremanao) ^24^^,^^10^^,^^11^^,^^24^_;_
	Além das tarefas supracitadas, as doulas da morte também realizam seus servinos em comunidades compassivas:
	Ajudam a estabelecer redes de apoio para o paciente ^2^^-^^4^^,^^10^^,^^11^^,^^23^;
	Facilitam a comunicanao com servinos^2^^-^^4^^,^^10^^,^^23^ e apoios locais para aquisinao de equipamentos quando o paciente está em _domicilio_^2^^,^^3^^,^^10^^,^^23^_;_
	Organizam e estimulam as redes informais de cuidados (por ex: parentes mais próximos e amigos intimos)^10^^,^^23^^,^^25^;
	Coordenam horários de descanso e de visitas do paciente com a familia, amigos e pessoas da rede de apoio^2^^,^^9^^,^^23^^,^^24^;
	Mediam e apoiam dinámicas de comunicando e diálogo sobre as respostas dos membros da comunidade compassiva quando as realidades da escolha pessoal também se sobrepoem a realidade do fim de vida e da morte da pessoa em processo ativo de morte^3^^,^^10^^,^^23^.
	Espiritual Promovem apoio espiritual a pessoa que está morrendo e a familia^2^^,^^3^^,^^10^^,^^23^^,^^24^;
	Preparam o paciente para o morrer; para a morte propriamente dita e ajudam a familia no pós-morte^10^^,^^11^^,^^22^^,^^24^^,^^25^; Respeitam as crennas religiosas, espirituais e culturais do paciente em fim de vida ^2^^-^^4^^,^^9^^,^^11^^,^^21^^-^^25^;
	Auxiliam a pessoa que está morrendo a encontrar a paz e a aceitanao da morte^9^^,^^22^^,^^23^;
	Conversam com o paciente sobre os valores^9^^,^^21^, e as crennas espirituais^9^^,^^21^^,^^24^, resgatando a espiritualidade^9^^,^^21^^,^^24^e o sagrado^10^^,^^23^;
	Elaboram um memorial com fotos de aniversários; datas especiais e/ou comemorativas do paciente com os familiares e amigos, incluindo mensagens de afeto^2^^,^^3^^,^^24^;
	Estimulam o paciente a elaborar o seu legado de vida^2^^,^^3^^,^^10^^,^^11^^,^^23^^,^^24^: a escrever cartas^2^^,^^3^^,^^9^^,^^10^^,^^24^; a organizar um álbum de fotos^2^^,^^3^^,^^24^; a gravar videos^2^^,^^3^^,^^10^^,^^24^; a escrever sua biografia^2^^,^^3^; a gravar as últimas mensagens de vida^10^^,^^24^;
	Auxiliam o paciente a resolver pendencias psiquicas, sociais e espirituais, tais como: perdoar-se, pedir perdao as pessoas que magoou; encontrar alguém que deseja se despedir^9^^,^^23^^,^^24^; ajudam ao paciente a identificar como ela quer ser lembrada ^22^^,^^23^;
	Estimulam a familia a refletir sobre o processo ativo de morte do paciente^10^^,^^23^^,^^24^;
	Desenvolvem anoes relacionadas as últimas horas de vida, incluindo a despedida^10^^,^^23^^,^^24^ planejamento da vigilia entre os membros da familia, parentes e amigos^10^^,^^23^^,^^24^; facilitam conversas finais entre o paciente e membros mais importantes da familia^23^^,^^24^; elaboram rituais a beira leito de acordo com as crennas religiosas e/ou espirituais do paciente^10^^,^^24^
Informativa/Educacional	Explicam aos familiares sobre os cuidados e as necessidades atuais do paciente^10^^,^^23^^-^^25^;
	Explicam os termos médicos ao paciente, familiares e amigos^3^^,^^23^^,^^24^;
	Explicam a familia os sinais premonitórios do final de vida^10^^,^^25^ e do pós-morte^10^^,^^24^;
	Orientam sobre os cuidados voltados para os momentos que podem ser realizados pela família e pelos amigos no processo ativo de morte e nas últimas horas de vida (por ex: se despedir)^10^^,^^23^^-^^25^;
	Apoiam o paciente na comunicado antecipada aos familiares, aos parentes e aos amigos, as intenses e os desejos em relaao ao local de morte (por ex: morrer em casa) e os rituais de despedida (por ex: ser sepultado; ser cremado); com quem querem vivenciar o processo de morrer^2^^,^^3^^,^^9^^,^^23^;
	Auxiliam e apoiam no planejamento das Diretivas Antecipadas de Vontade (por ex: nao ser alimentado por sonda; nao ser intubado; nao ser ressuscitado; no desejo ou nao de doaao de órgaos) e Testamento Vital (por ex: ir a um cartório registrar as diretivas)^2^^-^^4^^,^^10^^,^^24^;
	Gerenciam Death Cafés^3^^,^^10^^,^^11^^,^^23^^-^^25^;
	Promovem educado para a morte^2^^,^^3^^,^^10^^,^^11^^,^^23^^-^^25^ (por ex: palestras, cursos, workshops, seminários, webnários em eventos públicos, em universidades, em comunidades, em programas de treinamento)^3^^,^^10^^,^^11^.

## Discussao

O trabalho das doulas da morte tornou-se mais evidente e crescente devido a defesa do movimento da boa morte, uma vez que provoca reflexóes acerca de atitudes e comportamentos da sociedade em geral em relagáo ao processo de morte e morrer, bem como os cuidados de fim de vida^3^^,^^4^^,^^9^^-^^11^'^21^'^23^

Os papeis das doulas da morte apresentam, em seu escopo filosófico, uma visáo integral e holística do cuidado humano, abrangendo as dimensóes biopsicossocial, espiritual e educativa^2^^-^^4^^,^^9^^-^^11^^,^^21^^-^^25^, e, em seu escopo prático, realizam cuidados práticos e náo clínicos baseados na ortotanásia e kalotanásia, como se pode constatar no Quadro 2

As doulas da morte prestam seus servidos nas tres fases do processo de morte e morrer: a fase pré- morte, ou seja, aquela que vai desde o diagnóstico da doenga; a fase da morte, que é caracterizada pelo processo ativo da doenga, e, a fase pós-morte, que vai desde o óbito e se estende até o luto dos familiares^2^^-^^4^^,^^9^^-^^11^^,^^21^^-^^24^

A variedade de papeis nos servidos das doulas da morte tem como pedra basilar os cuidados religiosos, humanísticos e de saúde existentes para o paciente e seus familiares^2^^-^^4^^,^^9^^-^^11^^,^^21^^-^^24^. A diversidade de papeis e a inexistencia de um consenso de práticas recomendadas ocasionam conflitos entre as doulas da morte e outros profissionais de saúde, o que dificulta o trabalho^3^^,^^4^^,^^10^^,^^11^^,^^21^^,^^22^

As doulas da morte oferecem várias vantagens na melhoria do processo de morte e morrer: na área assistencial, ofertam cuidados individualizados, com abordagem humanística e centrada na pessoa. ^2^^-^^4^^,^^11^; trabalham diuturnamente, em toda rede de atengáo a saúde - seja comunidades compassivas ^2^^-^^4^^,^^10^^,^^11^^,^^21^, domicilios^2^^-^^4^^,^^10^^,^^11^^,^^21^, hospitais^2^^-^^4^^,^^10^^,^^11^^,^^21^^,^^23^, hospices^10^^,^^21^, instituyes de longa permanencia^9^^,(^^10^^,^^21^^-^^24^, casas de apoio^24^, aldeias indígenas 25 e presidios ^23^, voluntariamente ou vinculadas a algum servido de saúde ou previamente contratadas pela familia (de forma autónoma), como se pode constatar no Quadro 1; e na área educacional, gerenciam Death Cafés^3^^,^^4^^,^^10^^,^^21^^,^^23^ e promovem educagáo para a morte ^2^^-^^4^^,^^10^^,^^21^^,^^23^, como se pode observar no Quadro 2

O trabalho da doula no final da vida concentra-se em auxiliar o paciente a resgatar o sentido e o significado da vida durante o processo de morrer^2^^-^^4^^)),(^^9^^-^^11^^,^^21^^-^^25^, acompanhando-o e apoiando-o desde o diagnóstico até a morte propriamente dita, bem como apoiam familiares no luto^2^^-^^4^^,^^10^^,^^21^^-^^24^.Estudo^21^ descreve um modelo de atuagáo de doulas da morte para comunidades compassivas, mas que pode ser utilizado para quaisquer cenários da rede de atengáo a saúde e para quaisquer tipos de público-alvo. Nesse modelo, as doulas acompanham, auxiliam e mediam conversas entre paciente e familiares.

As doulas da morte náo só complementam os cuidados prestados pela equipe multiprofissional, mas se centram na singularidade do paciente e seus familiares mais próximos, como também procuram tornar a morte e o processo de morrer menos clínico, mais pessoal e mais significativo^2^^-^^4^^,^^10^^,^^11^^,^^21^^,^^23^.

Os servidos das doulas da morte náo buscam interferir ou alterar a estrutura de atendimentos existentes; em vez disso, concentram-se em dar continuidade e integrado dos cuidados ao longo da trajetória da morte dentro dos limites estruturais existentes, preenchendo lacunas e complementando o trabalho das equipes de cuidados paliativos^22^.

Estudos^2^^,^^9^^,^^10^ ressaltam que as primeiras doulas da morte foram enfermeiras com longa experiencia profissional, contudo, outros estudos salientam que qualquer pessoa - profissional de saúde^2^^-^^4^^,^^9^, ^10^^,^^22^^-^^24^^)^ ou náo^4^^,^^21^^,^^23^^,^^25^pode realizar os papeis de doulas da morte, desde que devidamente treinadas^22^, como pode ser visto no Quadro 1.

É a partir desta conjuntura que o movimento da doulas da morte apresenta vários desafios: a falta de um órgáo regulamentador que padronize as boas práticas de doulagem, supervisáo, fiscalizado e tabela de honorários; diversos programas de treinamento, com variado de conteúdos, metodologias, formatos (presencial e virtual), carga horária teórica e/ou prática em sua formado^2^^-^^4^^,^^9^^,^^11^^,^^22^^,^^24^. Por náo haver padronizagáo nos conteúdos formativos, existe uma preocupado e cautela de outros profissionais, público-alvo e usuários quanto a competencia, qualidade dos servidos e condutas éticas envolvendo o profissional doula^2^^-^^4^,^9^^,^^11^^,^^22^.

Somente um estudo^25^ descreveu o conteúdo programático de um curso de treinamento, a saber: cuidados paliativos (definido e principios); cuidados no processo de morte e morrer; comunicado em fim de vida; processo de enlutamento; diretivas antecipadas de vontade; intervendes médicas e náo médicas e estratégias para cumprir os desejos do paciente.

Existem programas de treinamento abrangentes, contínuos e com supervisáo nos Estados Unidos, Austrália, Canadá e Reino Unido^2^^-^^4^^,^^9^^-^^11^^,^^21^^-^^24^, e, grupos de apoio para as doulas partilharem suas experiencias, informarem os cuidados prestados e dirimirem suas dúvidas^24^.

Em relado a remunerado, todos os artigos^2^^-^^4^^,^^9^^-^^11^^,^^21^^-^^24^ da amostra mencionam que as doulas prestam servidos mediante pagamento ou realizado voluntariamente. Somente um estudo 4 revelou que as doulas da morte cobram por consultoria ou por hora de servido ou estabelecem pacotes de servidos (de 20h ou 30h ou 40h). Alguns colaboradores sentem-se envergonhados em cobrar os seus honorários e outros gostariam de realizar a transido profissional para trabalhar somente como doula.

Outro estudo^10^ especificou que algumas doulas náo cobram por seus servidos, mas caso os familiares queiram remunerar, o valor fica a critério da família, e, outras doulas se recusam em receber quaisquer remuneragóes, uma vez que seria contraditório a filosofia do trabalho.

Assim, acredita-se que a regulamentagáo e a padronizagáo de papeis e práticas das doulas da morte facilitariam o processo de comunicagáo com outros profissionais e, dessa forma, potencializariamo reconhecimento público dessa nova abordagem de cuidados em fim de vida, abrindo o caminho para a legitimagáo da profissáo^2^^,^^3^^,^^4^^,^^11^.

## Conclusao

A doula da morte é um (a) colaborador (a), cujo objetivo é melhorar a qualidade de morte no processo de terminalidade de pacientes, de forma a proporcionar uma ‘boa morte'.

Dentre a diversidade de papeis, as doulas prestam cuidados práticos e nao clínicos durante todo o processo de morrer, morte, pós-morte e luto de pacientes e seus familiares, bem como promovem educado para a morte.

Possíveis barreiras no movimento de doulas da morte incluem a inconsistencia nos programas de treinamento existentes e a ausencia de um órgáo regulamentador para a supervisáo da prática e a padronizado de honorários.

A atuagáo das doulas da morte, no Brasil, ainda, nao é regularizada, todavia, já existe um movimento crescente para a criado de uma associagáo, a partir da uniáo das doulas formadas pela AmorTser, empresa pioneira no Brasil e na América Latina, reconhecida legalmente, desde 2018.

As limitagóes do estudo estáo relacionadas a quantidade exigua de pesquisas, com desenhos metodológicos, em sua maioria, derivando de investigagóes qualitativas e/ou ensaios narrativos, por esse motivo, nao se pode generalizar os achados para outros países e outros contextos.

Se fazem necessárias novas investigagóes sobre perspectivas e experiencias de pacientes e familiares, bem como a percepgao de profissionais de saúde sobre a integragao das doulas da morte na rede de atengao a saúde.

Até o presente momento, nao se encontrou nenhuma publicagao científica brasileira indexada em bases de dados nacionais e internacionais sobre o movimento das doulas da morte no Brasil e nem na América Latina.
